# Future Match Making: When Pediatric Oncology Meets Organoid Technology

**DOI:** 10.3389/fcell.2021.674219

**Published:** 2021-07-13

**Authors:** Virginie Barbet, Laura Broutier

**Affiliations:** Childhood Cancer & Cell Death (C3), Université Claude Bernard Lyon 1, INSERM 1052, CNRS 5286, Centre Léon Bérard, Centre de Recherche en Cancérologie de Lyon (CRCL), Lyon, France

**Keywords:** organoids, tumoroids, cancer, modeling, genetic engineering, heterogeneity, plasticity, pediatric cancer and oncology

## Abstract

Unlike adult cancers that frequently result from the accumulation in time of mutational “hits” often linked to lifestyle, childhood cancers are emerging as diseases of dysregulated development through massive epigenetic alterations. The ability to reconstruct these differences in cancer models is therefore crucial for better understanding the uniqueness of pediatric cancer biology. Cancer organoids (i.e., tumoroids) represent a promising approach for creating patient-derived *in vitro* cancer models that closely recapitulate the overall pathophysiological features of natural tumorigenesis, including intra-tumoral heterogeneity and plasticity. Though largely applied to adult cancers, this technology is scarcely used for childhood cancers, with a notable delay in technological transfer. However, tumoroids could provide an unprecedented tool to unravel the biology of pediatric cancers and improve their therapeutic management. We herein present the current state-of-the-art of a long awaited and much needed matchmaking.

## Introduction

Pediatric cancers differ quite significantly from adult cancers. First, unlike adult cancers, childhood cancers are rare, affecting approximately 15 per 100,000 children annually (Waldron et al., 2010). Second, whereas adult tumors are most commonly carcinomas derived from highly differentiated epithelial tissues, such as in breast, lung, colon, and prostate cancers, pediatric cancers encompass a heterogeneous set of diseases that can be broadly subclassified into leukemias, lymphomas, brain and non-central nervous system tumors, sarcomas, and additional rare cancers (Steliarova-Foucher et al., 2005). Third, arising in the context of actively growing tissues, childhood cancers are emerging as diseases of dysregulated development, corroborating the statement that “oncogeny is blocked ontogeny” already summarized by Van R. Potter in the 1960s, whereas adult cancers are often associated with lifestyle (Khan et al., 2010; Willyard, 2011). Consequently, the genomic landscape of childhood cancers also differs. Not only is the mutational burden lower in childhood malignancies compared with adult cancers, but the types of alterations and mutated genes also differ. Indeed, rather than numerous mutational “hits” frequently observed in adult cancers, recent sequencing studies have revealed that pediatric cancer-driving point mutations are enriched in genes that encode epigenetic machinery (Gröbner et al., 2018; Ma et al., 2018). Moreover, fusion oncoproteins are particularly prevalent among childhood cancers (Gröbner et al., 2018; Ma et al., 2018; Filbin and Monje, 2019). They result from translocation juxtaposing oncogenes with partner genes that often activate genes crucial to development, such as the paired box (Pax) genes encoding a family of transcription factors that orchestrate complex processes of lineage determination in the developing embryo (Blake and Ziman, 2014). Interestingly, both these driver point mutations and oncogenic fusion events are largely specific to individual cancer types in which they arise (Gröbner et al., 2018; Ma et al., 2018). Another feature of pediatric cancers is that a relatively high percentage of patients (∼8–10%; Gröbner et al., 2018; Ma et al., 2018; Savary et al., 2020) carry an unambiguous germline mutation that predisposes them to developing cancer. Finally, childhood cancers are often cited as the modern success story of medical research owing to the advent of chemotherapy. Cure rates for childhood cancers have evolved from <25% in the pre-chemotherapy era to about 80% (Gatta et al., 2009) in recent decades (Pui et al., 2011; Saletta et al., 2014). However, we need to keep in mind that pediatric cancer is still the leading cause of death by disease for children in western countries, with 20% of children not responding or relapsing after first-line treatment, and with a very poor outcome due to the absence of efficient second-line therapies (Steliarova-Foucher et al., 2005). Moreover, many children who survive cancer suffer from long-term sequelae including organ toxicity, mental disabilities, and secondary cancers, and some pediatric cancers, such as diffuse intrinsic pontine glioma, are still uniformly fatal (Landier et al., 2015; Vanan and Eisenstat, 2015).

The differences arising between pediatric and adult tumors emphasize the need for considering pediatric cancers separately, and demonstrate the requirement for distinct therapeutic approaches compared with adult cancers. However, the *in vivo* testing of such novel therapies remains limited in children for ethics reasons, and the reliance on cancer models reconstructing tumoral heterogeneity is therefore primordial for understanding pediatric cancer biology and response to therapies. Over the past 20 years, advances in stem cell biology and *in vitro* three-dimensional (3D) culture technologies have heralded a revolution in biology and medicine. A major recent step in this revolution has been the development of methods to generate, under controlled cultured conditions, 3D structures, known as organoids (Clevers, 2016). Among multiple organoid applications, the establishment of cancer organoids (i.e., tumoroids) has recently emerged as a prominent tool to enhance our understanding of human cancers by faithfully mimicking *in vitro* both inter- and intra-tumoral heterogeneity (Gao et al., 2014; Boj et al., 2015; Matano et al., 2015; Broutier et al., 2017; Shimokawa et al., 2017; Fumagalli et al., 2020; Jacob et al., 2020; Karakasheva et al., 2020). In this review, we present some of the models used in the pediatric cancer field over the past century. Moreover, we define and describe current cancer organoid models and provide an overview of their use and importance in basic and translational research. Many excellent reviews have described the variety of organoid models highlighting their relevance for cancer modeling (Clevers, 2016; Drost and Clevers, 2018; Smith and Tabar, 2019; Tuveson and Clevers, 2019), and we will herein focus on why and how these tumoroids could also provide an unprecedented tool to unravel the biology of pediatric cancers, in particular, to explore the role of dysregulation of epigenetic processes involved in cell specification and plasticity in pediatric oncogenesis. Finally, we offer concluding remarks and perspectives in line with these findings, to draw attention to the scarcity of exploitable data and data sharing in the field of pediatric cancer.

## Historical Overview of Pediatric Cancer Models

**FIGURE 1:**
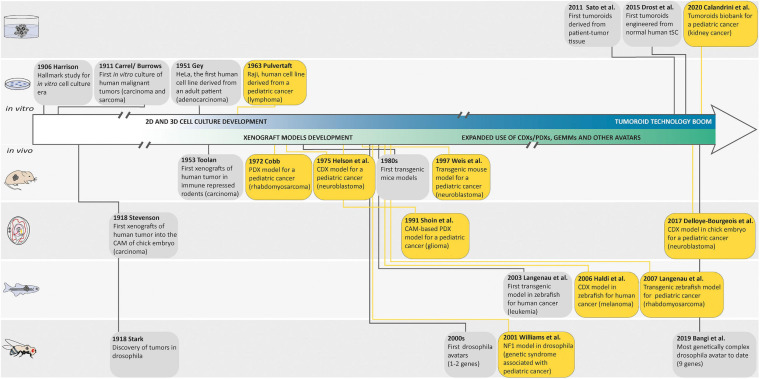
Landmark studies in cancer model discovery. The history of cancer models can be retraced back to the early 1900s, when Harrison described the first tissue culture technique and when Carrel and Burrows defined a basic protocol to standardize the *in vitro* culture of human malignant tumors. Thereafter, the development of these cell culture techniques generated the first human cell line derived from a patient (HeLa) in 1951 and the Raji cell line, as one of the first pediatric cancer cell lines in 1963. In parallel with these *in vitro* models, many animal cancer models emerged following the discovery of tumors in drosophila in 1918, the first xenografts of human tumor in chorioallantoic membrane (CAM) of chick embryo in 1918, in immune-repressed rodents in 1953, or in zebrafish in 2006, and the generation of genetically engineered animal cancer models. Later, in order to recreate a cancer model closely mimicking the histological complexity and genetic heterogeneity of human cancers, the first tumoroids derived from a patient tumor tissue were described by Sato et al. in 2011. The first tumoroids were then engineered from normal human tissue-specific SCs and the generation of an organoid biobank for a pediatric cancer was described. Yellow boxes highlight pediatric cancer models.

The use of *in vitro* cancer cell lines and animal model systems in cancer research during the late 20th and early 21st centuries has been successful in many areas, such as improving our understanding of oncogenic signaling pathways, identifying potential drug targets, or guiding the design of candidate drugs. Historically, the development of these conventional cancer models has progressed along a common discovery pipeline (Figure 1).

Most of our current understanding of cancer and its hallmarks is based on the establishment of long-term *in vitro* cultured tumor cell lines. It is commonly admitted that tissue culture has arisen around the turn of the 20th century with the hanging drop tissue culture technique developed by Ross Harrison for frog neurons (Harrison, 1906). Several years later, Carrel and Burrows defined a basic protocol to standardize the *in vitro* culture of cells from different tissues of origin. In particular, sarcoma and carcinoma samples that were obtained from rats, dogs, and humans were cultured *in vitro* while using horse or bovine plasma (Carrel and Burrows, 1911). The development of techniques and media for cell culture subsequently improved, and in 1951, Gey established the first continuous, and internationally acknowledged, human cancer cell line derived from an adenocarcinoma of the cervix (HeLa cell line established from a biopsy of Henrietta Lacks; Gey et al., 1952). A decade later, Pulvertaft established the Raji cell line, as one of the first pediatric cancer cell lines derived from an 11-year-old African Burkitt lymphoma patient (Pulvertaft, 1964). Since then, hundreds of cancer cell lines have been established and propagated *in vitro*. However, 2D cancer cell lines have many limitations, such as non-physiological interactions between the cellular and extracellular environments, changes in cell morphology and polarity, and lack of reflection of the genetic diversity within a whole tumor. Indeed, cancer cell lines, after several passages, lose many features of their original *in vivo* state, notably through selection and expansion of clones capable of growing in non-physiological conditions (Lee et al., 2006). Moreover, cells cultured *in vitro* for several passages display substantial and unpredictable genetic changes (Lee et al., 2006). These disadvantages led to the creation of models that are more closely able to mimic conditions *in vivo*.

First, 3D cell culture methods were developed to recreate a more physiologically relevant environment. In contrast to 2D resulting in a monolayer cell expanding on a flat surface of glass or commercial polystyrene plastic flasks for tissue culture, 3D cell cultures usually promote the formation of cellular aggregates. Throughout the 20th century, researchers working toward generating organs *in vitro* from dissociated cells helped this field to evolve rapidly [for an overview, see Lancaster and Knoblich (2014) and Simian and Bissell (2017)], and spheroids, defined as 3D cellular aggregates and obtained from a large diversity of cell types, such as immortalized cancer cell lines or primary tumoral cells, are currently the most common way to culture cancer cells in 3D (Edmondson et al., 2014; Costa et al., 2016). These 3D spheroids can be cultured using a wide range of methods relying on the use of a supporting scaffold or the intrinsic capacity of cells to self-assemble into clusters when cultured on non-adhesive materials, under surface tension, and gravitational force (e.g., hanging drop techniques) or constant circular rotation of vessels (e.g., spinner culture; Edmondson et al., 2014; Costa et al., 2016). In all cases, these techniques facilitate cell–cell and/or cell–matrix interactions to overcome the limitations of traditional monolayer cell culture (Ryu et al., 2019). Although these 3D cultures have a greater physiological relevance than 2D cultures and have drawn increasing interest in drug discovery due to their evident advantages in providing more predictive data for *in vivo* tests, the microenvironment is often not maintained, and neither are long-term propagation ability nor genetic heterogeneity and stability. Hence, the need for/use of other cancer models still remains.

Second, both cancer cell lines and surgically derived primary clinical tumor samples have been grafted into animals, predominantly rodents. These models are known as cell line-derived (CDX) and patient-derived (PDX) xenografts, respectively. In such models, tumor architecture and the relative proportion of cancer cells and stromal cells are maintained to a large extent, which yields better resemblance to the original tumor compared with *in vitro* cancer cell lines. In 1953, a PDX hallmark study by Toolan (1953) showed that it was possible to grow human tumor cells in *x*-irradiated rodents. In that experimental setup, 101 human tumors implanted in cortisone-treated *x*-radiated rodents, 90% survived and proliferated for 12–20 days. Considering pediatric solid tumors, a delay of almost 20 years saw the first attempt at xenotransplantation by Cobb et al. in 1972 of a rhabdomyosarcoma specimen into thymectomized hamsters, which were additionally treated with antithymocyte serum. In that study, PDX resulted in limited local tumor growth in two-fifths of the animals and in the development of lung metastases in one-third of the animals (Cobb, 1972). Thereafter, the first successful CDX was reported in 1975 by Helson et al. In this study, human neuroblastoma cell lines (SK-N-SH and SK-N-MC) were injected into immunodeficient Swiss Webster mice. Tumor growth developed at the injection site within 8 to 21 days, and the tumors were histologically identical to the original tumor with evidence of morphological differentiation (Helson et al., 1975). Later, the development of immunocompromised mice resulted in improving the success rate of engraftment and the establishment of CDX and PDX models for a broad variety of adult and pediatric cancers (Shultz et al., 1995, 2005; Traggiai et al., 2004; Drake et al., 2012). In the past 50 years, many studies demonstrated the value of CDX and PDX in rodents as preclinical models to better understand cancers, develop novel treatments, predict clinical response in patients, and unravel biomarkers for drug sensitivity and resistance (Houghton et al., 2007; Monsma et al., 2014; Rosfjord et al., 2014; Gao et al., 2015; Geier et al., 2015; Nicolle et al., 2016; Townsend et al., 2016; Mohamed Suhaimi et al., 2017; Stewart et al., 2017; Brabetz et al., 2018; Rokita et al., 2019).

Rodents are not the only animals used to perform CDX and PDX models. The first applications of the chick embryo and chorioallantoic membrane (CAM) in oncology research were announced more than a century ago (Murphy and Rous, 1912). In 1914, Murphy observed that transplants of rat tissue could grow on the vascular CAM of chicks up until developmental day 18 (Murphy, 1914), which demonstrated the natural immunodeficiency of the developing chick, rendering it amenable to tumor xenografting (Stevenson and Wood, 1918). Since then, chicken CAM assays have been largely used for modeling tumor angiogenesis, cancer metastasis, or drug testing. For example, in the field of pediatric oncology, Shoin et al. (1991) assessed CAM-based PDX model efficacy for two drugs used in the treatment of malignant glioma, ependymoma, and medulloblastoma, and found a high degree of positive association between the chick embryo assay and clinical outcome. Moreover, Ribatti et al. (2001) developed a CAM-based PDX model for neuroblastoma and noted that a high vascular index was correlated with poor prognosis, making the CAM particularly appropriate to investigate neuroblastoma growth with its metastatic process, and to use as a platform for anti-metastatic drug testing (Ribatti and Tamma, 2018; Swadi et al., 2018; Pawlikowska et al., 2020). Of note, in 2017, Delloye-Bourgeois et al. (2017) developed a model in which human neuroblastoma cell lines were grafted directly into the chick sympatho-adrenal neural crest, providing an innovative and relevant model to uncover the molecular players involved in the onset of metastatic neuroblastoma. Zebrafish embryo and larvae are also good transplant recipients, as their immune system does not fully mature until 4 weeks of age. In 2006, the first human cancer cell xenograft in zebrafish larvae was reported for melanoma (Haldi et al., 2006). 10 years later, proof-of-concept studies were published showing the development of PDXs in zebrafish larvae from solid tumors (Mercatali et al., 2016; Fior et al., 2017), suggesting that zebrafish larvae xenografts could be a promising *in vivo* screening platform for drug testing and precision medicine. However, the lower temperatures required for larval growth (28–35°C) eventually kill the human cells, narrowing down the relevance of this model. In that context, optically clear zebrafish strain that lacks T and B lymphocytes and natural killer cells, and grows at 37°C represents an exciting recent advance to allow long-term xenotransplantation of cancer cell lines and patient-derived cancer cells into adult zebrafish (Yan et al., 2019) and will have to be explored in the field of pediatric cancers. Albeit, immunocompromised models are still not ideal as many cancer patients retain functional immune systems prior to therapy.

In an insightful alternative approach to tumor transplant models for biological and therapeutic investigations, the generation of genetically engineered mouse models (GEMMs) emerged in the 1980s based on an increasing understanding of the genetic aberrations underlying tumorigenesis [for an overview, see Hanahan et al. (2007)]. A wide range of GEMMs for adult cancers were developed in the following 10 years. Eventually, in 1990, Windle et al. (1990) developed one of the first pediatric cancer GEMMs using the simian virus 40 (SV40) large T antigen (Tag) gene cohybridized with the luteinizing hormone β-subunit (LHβ) gene as promoter. This model produces heritable ocular tumors with histological, ultrastructural, and immunohistochemical features identical to those of human retinoblastoma. Later in 1997, Weiss et al. (1997) developed the most widely used GEMMs for neuroblastoma research. This transgenic mouse overexpresses MYCN through a tyrosine hydroxylase promoter (TH-MYCN) and was the first model used to demonstrate that MYCN amplification can drive neuroblastoma development, highlighting the MYCN pathway as a potential therapeutic target. In contrast to PDX and CDX models, GEMMs display *de novo* tumorigenesis in a fully immunocompetent host environment. Moreover, GEMMs recapitulate molecular and histopathological features of human cancer and can model metastatic disease. Thus, they reproduce both cancer cell-intrinsic and cell-extrinsic components, and can successfully be used to validate new oncogenic pathways, drug targets, and assess therapeutic efficacy including in the case of immunotherapies.

Even though mouse is the most common system used for the generation of genetically engineered models for cancer research, others research animals can be efficiently genetically modified and have contributed greatly to our understanding of human malignancies. Zebrafishes are relatively easy to genetically engineer by microinjection at the one-cell stage embryo. They present a significant conservation of human cancer-associated genes. Moreover, they develop cancers that are histologically and genetically close to those of humans. In 2003, Langenau et al. (2003) demonstrated that expression of the mouse oncogene Myc under the zebrafish recombination activating gene 2 (rag2) promoter resulted in the rapid onset of adult leukemia that emerge from the thymus. Since then, more than 50 genetically engineered zebrafish models of human cancers have been established and characterized, and have been shown to closely mimic their human counterparts at the histological and/or genomic levels. They helped to accelerate the discovery of new mechanisms driving human cancers and identify new drugs for clinical trials. For example, in 2007, the most studied zebrafish model of embryonic rhabdomyosarcoma (ERMS), a pediatric sarcoma exhibiting skeletal muscle features, was developed using a rag2 promoter to drive the expression of a constitutively active *kras*^*G12D*^ gene in muscle satellite cells (Langenau et al., 2007). Gene set enrichment analysis and RNA *in situ* hybridization studies using clinical markers of human RMS validated that the genomic landscape of zebrafish tumors closely resembles human ERMS (Langenau et al., 2007). Moreover, this zebrafish ERMS model has been used to identify potential genes that influence the behavior of tumor-propagating cells during ERMS initiation, and to test the efficacy of drugs (Le et al., 2012). For an overview on how zebrafish pediatric cancer models have and could in the future advance the field of pediatric cancers as a preclinical model please refer to Casey and Stewart (2020). The fruit fly, *Drosophila melanogaster*, is used as a model organism to study disciplines ranging from development to disease. Notably, conservation of signaling pathways controlling cell growth, differentiation, and invasion between humans and flies, and the availability of powerful genetic tools has made *Drosophila* a useful model organism to study cancer biology. From the initial studies of Mary Stark discovering tumors in *Drosophila* and providing the experimental support for the theory of cancer as a disease of the chromosomes in 1918 (Stark, 1918), through the breakthrough research of Elizabeth Gateff on tumor suppressor genes (Gateff and Schneiderman, 1967, 1969, 1974) to the revival of the fly model for cancer studies at the beginning of the new century with genetically engineered *Drosophila* developing human cancers such as thyroid, lung, prostate, gut, or brain models, *Drosophila* models have proven to be a very powerful model to study cancer. For example, *Drosophila* has been successful used to study Neurofibromatosis 1, a genetic syndrome with highly elevated risk of associated childhood cancer. The mutant *Nf1* fly provided some of the first evidence that dysregulation of Ras pathway signaling is a major cause of Neurofibromatosis 1 (Williams et al., 2001), and this result was further supported by Barkan et al. (2006), who in 2006 demonstrated the use of a Ras inhibitor as a potential drug for the treatment of NF1. More recently, using *Drosophila*, Narbonne-Reveau et al. (2016) described a neural stem cell-encoded clock that delineates an early window of malignant susceptibility during early development underlining the importance of deciphering temporal specification mechanisms in the nervous system to help in identifying the cell types and gene networks at the origin of pediatric neural cancers. As a leading example of the power of *Drosophila* in oncology, flies, known as avatar flies, are currently being genetically engineered to carry the specific mutations of a cancer patient, and are used to define specific anti-cancer drug cocktails for personalized medicine and could hold great promise for pediatric cancers (Bangi et al., 2019).

In the past century, conventional models presented above have offered useful insights into cancer research that have guided the design of innovative candidate drugs. However, many of these drugs fail in human clinical trials because of either ineffectiveness or unbearable side effects. This can, at least in part, be explained by the fact that the histological complexity and genetic heterogeneity of human cancers are typically not reflected in those conventional models. While cell lines are constitutive to many types of experimental work, they tend to drift away from the genomic features or growth characteristics of parent tumors over time, a widely recognized limitation. Likewise, despite the undeniable importance of animal models, they also have some drawbacks. In particular, their genomic and immune profile does not match complex tumor–host interactions. Indeed, a number of biological phenomena that are specific to humans are not amenable to being reproduced in other animals. Therefore, additional disease modeling strategies that bridge the gap between animal models and human beings and mimic the histological complexity and genetic heterogeneity of human cancers are needed to complement existing techniques in cancer research. *The emergence of the organoid technology based on stem cell and 3D culture approaches has therefore received worldwide attention as having the potential to overcome some conventional cancer model limitations.*

## Organoids: Innovative Tools in Cancer Research

Classical models have shown their limits in understanding the molecular basis of childhood and adolescent cancers and in modeling the effects of new therapies. In recent years, the epigenetic component of pediatric cancers, their strong developmental valence, and the impact of functional heterogeneity and plasticity on resistance to treatments have emerged as key levers to be understood in a specific way to improve their therapeutic management. In this context, the recent successes achieved for certain adult cancers suggest that organoids undeniably have the characteristics to face these challenges in pediatric oncology.

### Organoid Definition and Generation

#### A Need for Definition

Over the past 20 years, advances in stem cell biology and *in vitro* 3D culture technologies have heralded a revolution in biology and medicine. A major recent step in this revolution has been the development of methods to generate, under controlled cultured conditions, 3D structures, known as organoids (Bartfeld and Clevers, 2017). The word organoid initially defined a series of cell culture techniques that are not necessarily a single technique (Lancaster and Knoblich, 2014). However, since organoid technology is becoming an independent research tool, a precise definition is necessary. Based on grammar, -oid is a suffix meaning “resembling,” used in the formation of a noun, which *per se* implies an incomplete or imperfect resemblance to what is indicated by the preceding element. In essence, organoid thus means “resembling an organ.” Several elements have been added in the past decade to refine this broad definition. First, one feature is common to all organoids: they are derived from stem cells, pluripotent stem cells (PSCs), or tissue-specific stem cells (tSCs). Second, organoids have to contain several cell types that develop from these stem cells and self-organize through cell sorting and spatially restricted lineage commitment as observed *in vivo* during organogenesis or tissue regeneration. Finally, organoids have to exhibit at least some organ functionalities of the modeled tissue. Hence, we can define organoids as *in vitro 3D cellular clusters grown from stem cells, in which cells self-organize into progenitors and more differentiated organ-specific cell types, which recapitulate at least some functions of the organ of interest* (Lancaster and Knoblich, 2014; Clevers, 2016). In 2009, a landmark study from Sato et al. showed that single leucine-rich repeats containing G protein-coupled receptor 5 (Lgr5)-expressing adult intestinal stem cells in appropriate culture conditions could form 3D intestinal structures faithfully mimicking their *in vivo* counterpart. Indeed, these 3D cultures self-organized and differentiated into crypt–villus structures (Sato et al., 2009). This work set the scene for many subsequent organoid models from various sources. [For an overview see Kim et al. (2020)].

#### How Are Organoids Generated?

**FIGURE 2:**
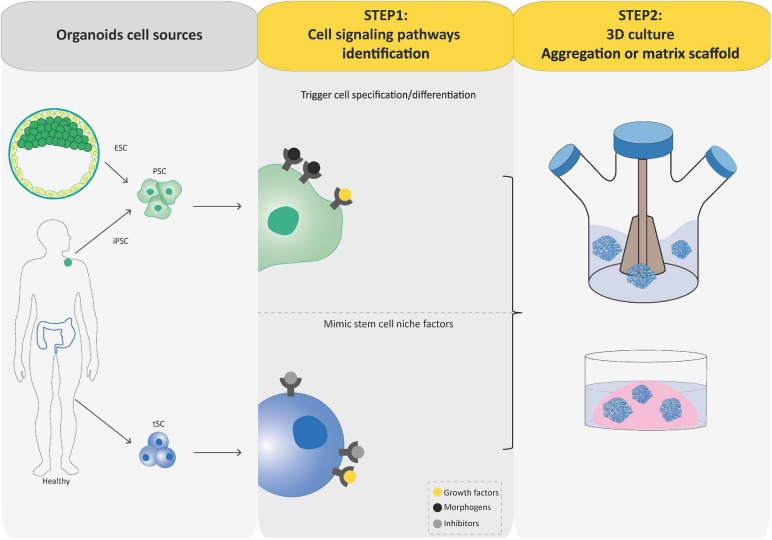
Flowcharts of the establishment of pluripotent stem cell (PSC)-derived and tissue-specific stem cell (tSC)-derived organoids. Organoids can be established from PSCs (iPSCs and ESCs) or tissue-specific SCs (tSCs; left-hand box). Two key steps are involved in PSC-derived and tSC-derived organoid production. First (central box), the identification of crucial cell signaling pathways allowing directed differentiation (combinations of morphogens and growth factors) and/or to establish a permissive environment for the stem cell culture by mimicking their *in vivo* niche (specific growth factors and inhibitors). Second (right-hand box), cultures are grown so as to favor their expansion in three dimensions, which is achieved either by aggregating cells into 3D structures or by embedding the cultures into a 3D matrix scaffold.

Regardless of the stem cell source, the process to generate organoids involves two key steps relying on our understanding of stem cell physiology including their ability to self-renew and generate differentiated cells. First, crucial signaling pathways regulating self-renewal, proliferation, quiescence, cell-death, and cell-lineage commitment are inhibited or activated using commercially available signaling inhibitors and morphogens, or conditioned media in order to establish a permissive environment for the stem cell and its progenies to expand and acquire correct identity during spatially restricted lineage commitment occurring in organoids. The idea here is to exploit developmental processes for PSC-based organoid establishment and mimic the *in vivo* stem cell niche present during physiological tissue self-renewal or during damage repair for tSC-based organoid establishment (Figure 2). Second, cultures are grown in three dimensions, which is achieved either by aggregating cells into 3D structures and/or by growing them within or on 3D supporting matrix scaffold (biological or synthetic hydrogels that resemble the natural extracellular matrix; Figure 2). Usually, PSCs undergo stepwise differentiation protocols, in which the timing, concentration, and combination of specific cues is crucial in order to progress to defined differentiation states within developmental lineages. During this process, cells aggregate to form organoids that faithfully mimic the desired organ (Spence et al., 2011; Lancaster et al., 2013; Dye et al., 2015; Trisno et al., 2018). In contrast to these sophisticated processes, tSC-derived organoids can be generated directly from a biopsy of the organ of interest, after mechanical and enzymatic dissociation and seeding of the cell suspension in an appropriate cocktail of growth factors, inhibitors, and often matrix scaffold to sustain differentiation and self-renewal (Sato et al., 2011; Karthaus et al., 2014; Linnemann et al., 2015; Sampaziotis et al., 2017; Turco et al., 2017; Hu et al., 2018; Sachs et al., 2019). This ability to self-renew is the main difference between PSC-derived organoids and tSC-derived organoids: tSC-derived organoids can be expanded for several passages, thus, forming the basis for the building of living biobanks for biomedical research (Boers et al., 2016).

Organoids do not require immortalization before *in vitro* culture and, thus, allow the study of close to native tissues in both their cellular identity, composition, and architecture, and are far more amenable to genome editing and high-resolution imaging than *in vivo* models. As such, they represent an important bridge between traditional 2D cultures and *in vivo* mouse/human models. The paucity of *in vitro* cancer models that faithfully mimic human cancers has impeded our full understanding of oncogenic processes and, consequently, our ability to anticipate therapeutic responses. Today, organoid technology appears as a straight-thinking approach for the creation of models that, as organoids of organs, closely recapitulate tumor cellular identity, composition, and architecture, and subsequent pathophysiological traits of natural tumorigenesis and metastasis *in vitro* (Muthuswamy, 2018).

### Organoid Technology and Cancers

#### A Need for Nomenclature, Definition, and Concept

In the past years, organoid technology has rapidly conquered the cancer research field as a powerfully exploitable tool to create innovative and robust models for both basic and translational cancer research applications. As for organoids, the recent extensive use of such models encourages a precise definition. In this review, we recommend the use of the term tumoroid. Indeed, following the logic of “organoid” meaning “resembling an organ,” tumoroid encompasses models aiming at reproducing a tumor in a dish and can be defined, by analogy to organoid, as *in vitro 3D cellular clusters derived from tumoral cells with stem cell-like properties (hereafter called cancer stem cells CSCs), in which cells spontaneously self-organize and recapitulate the histological, molecular and differentiation status of the tumor of interest.* They may be derived directly from tumoral tissues or generated by genetic engineering of PSCs or tSCs. Of note, the largely used term cancer organoid, i.e., “cancer resembling an organ” is incongruous and should be avoided. Moreover, cancer and tumor are often used as synonyms, but they do not always apply to the same thing. The term “tumor” is a commonly used term for a neoplasm, a type of abnormal and excessive growth, of tissue. Neoplasms may be benign or malignant (cancer). Tumor is therefore a general term that can refer to benign or malignant growths: as such, it has a broader definition than cancer and, thus, regroups more models based on the nomenclature defined above. Finally, we believe that the organoid technology applied to the field of cancer research should possess its own terminology since organs and tumors are very distinct entities, not governed by the same rules. We acknowledge that a better understanding of organ development and regeneration is key to unraveling tumor behavior that often results in the dysregulation or hijack of these physiological pathways, but we would like to emphasize here that dissecting tumor biology via this prism alone is a limiting approach considering the complexity of genetic and epigenetic intratumoral heterogeneity.

#### All Tumoroids Are Not Equal, but They All Have Assets to Herald a New Paradigm in Pediatric Cancer Modeling

**FIGURE 3:**
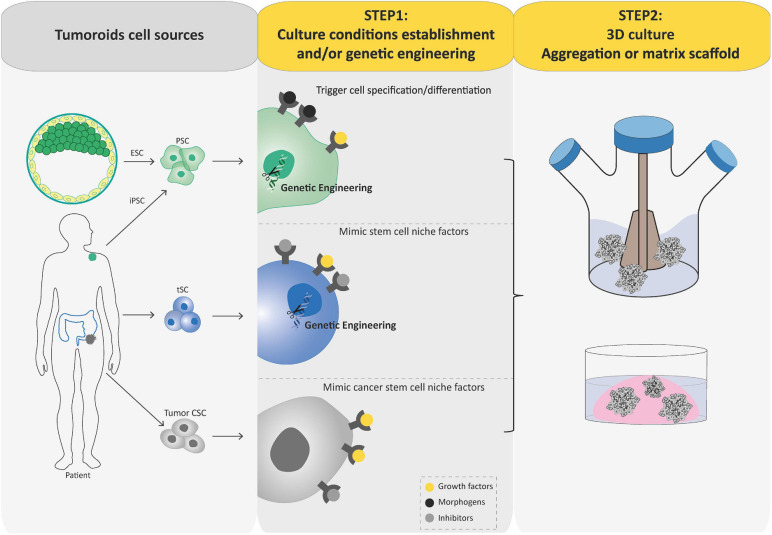
Schematic diagram depicting current methods for generating tumoroids. Tumoroids can be established from PSCs (iPSC and ESC), tissue-specific SCs (tSCs), and directly from tumor biopsies, resection, or fluids (left-hand box). Similar to organoids, two key steps are involved in tumoroid derivation. Following genetic engineering (for PSCs and tSCs) and directed differentiation in a specific cell lineage by morphogens (for PSCs), identification of key cell signaling pathways to mimic CSC niche factors (growth factors and inhibitors) allows tumoral cells to be cultured in three dimensions (aggregation or matrix scaffold; central and right-hand boxes).

Over the past decade, we have witnessed tremendous progress in SC technologies and 3D culture methods. Together with improved genome editing tools and differentiation protocols, these advances have facilitated the development of new models of human cancer (Hochedlinger and Plath, 2009; Artegiani et al., 2020; Gier et al., 2020), such as tumoroids that can be generated by engineering tumorigenic alterations of PSCs and tSCs or directly derived from tumoral tissues. Similar to organoids, the process of generating tumoroids can be summarized in two key steps (Figure 3). In this part, we aim to provide a concise but complete overview of these different tumoroid systems, their main attributes, and their key applications. For more details, please refer to excellent reviews on the topic (Drost and Clevers, 2018; Smith and Tabar, 2019; Lo et al., 2020).

##### Bottom–up approaches: from tumorigenic alterations to tumoroids

As cancer is thought to result from gradual accumulation of mutations in disease-driving genes, bottom–up tumoroid modeling mainly relies on introducing sequential oncogenic events to recapitulate tumoral initiation, evolution, and progression in specific organs. This strategy may be particularly appropriate for dissecting the mechanisms of tumor initiation and transformation in pediatric cancers, since it is known that their mutational burden is lower than that observed in adults and that one or two genetic oncogenic events may be sufficient to switch the cells to a tumor fate. Tumorigenic alterations, individually or in combination, can easily be introduced into either tSC- or PSC-derived organoids by different genome-editing tools such as CRISPR/Cas9-mediated gene editing and lentiviral transduction (Huang et al., 2015; Hockemeyer and Jaenisch, 2016; Sun et al., 2019) in order to obtain tumoroids. Overall, these tumoroid models build on tumorigenesis *de novo* by initiating genetic changes in normal cells, providing a unique opportunity for modeling oncogenic processes. Of note, cellular and molecular analyses in such models depend on comparisons with non-engineered cells, which, ideally, should be isogenic, i.e., derived from the same individual, so that they share all germline (but not somatic) variants.

Two landmark studies, starting from healthy human intestinal organoids, exploited CRISPR-Cas9 genome editing to introduce combinations of common colorectal cancer (CRC) driver mutations recapitulating the classical “Vogelgram” model (Fearon and Vogelstein, 1990; Drost et al., 2015; Matano et al., 2015). Interestingly, authors showed that CRC driver mutations support their growth as invasive adenocarcinoma *in vivo* into xenotransplanted mice, confirming the oncogenic transformation (Fumagalli et al., 2020). Additionally, they demonstrated that loss of *APC* and *TP53* are key drivers of aneuploidy and chromosome instability, two hallmarks of cancers (Drost et al., 2015). Tumorigenic engineering studies have rapidly progressed from early work in CRC, and today, the paradigm of oncogenic conversion of intestinal organoids has been enlarged to many prevalent solid tumor types. Thereby, it has shed light on the mechanisms through which certain cell types and differentiation states are more or less amenable to transformation by given mutations (Crespo et al., 2017). Reciprocally, bottom–up tumoroid models can also help to highlight how mutations can lead to a shift in cell identity (Friedmann-Morvinski and Verma, 2014). Moreover, bottom–up tumoroid modeling may provide important insight into tumor evolution, which may be difficult to apprehend in cancer cells due to the activity of many concomitant mutational processes, making it challenging to study the origin of mutational signatures. For instance, the origin of cancer-associated mutational signatures can be studied using CRISPR-Cas9 technology to delete key DNA repair genes in human organoids and whole-genome sequencing, as exemplified by Drost et al. (2017) Indeed, they found that depletion of *MLH1*, a key DNA repair gene, leads to the accumulation of mutations driven by replication errors in colonic organoids and accurately models the mutational profile observed in mismatch repair-deficient colorectal cancers. Moreover, the chemotherapeutic drug 5-fluorouracil (5-FU), commonly used for the treatment of solid cancers, was shown to accelerate tumor evolution (T > G substitutions) *in vitro* in intestinal organoids, corroborating results *in vivo* in colorectal and breast cancer patients who received 5-FU treatment (Christensen et al., 2019). These studies demonstrate the possibility of conducting longitudinal tumor evolution studies in human tumoroids and may provide novel insights into the deleterious side effects of chemotherapeutics and the increased risk of secondary cancers later in life. Along the same line, interplay between oncogenic alterations, and environmental and microenvironmental factors in the malignant process can be investigate in bottom–up tumoroids. For example, the culture conditions in which organoids are maintained have also been leveraged to uncover patient-specific aspects of cancer biology. Thus, CRC tumoroids established by sequential introduction of driver mutations result in a progressive loss of stem cell niche factor requirements during tumorigenesis *in vitro* and highlight microenvironmental dependencies of human CRC tumors according to their mutational pattern (Drost et al., 2015; Matano et al., 2015). Moreover, organoid co-cultures with pathogens can be used to model host–pathogen interactions as potential risk factors for cancer development. Some bacterial species are enriched in patients with colorectal cancers, and have been associated with such cancers without a direct demonstration. Recently, researchers exposed human intestinal organoids to genotoxic colibactin-producing *Escherichia coli* by repeated luminal injection. Whole-genome sequencing analyses revealed a distinct mutational signature [single thymine (T) deletion signature associated with an increase in T > N substitutions] that was absent from organoids injected with isogenic colibactin-free bacteria. The same mutational signature was detected in human colorectal cancer, suggesting a causal effect of a past exposure to bacteria carrying a colibactin-producing pathogenicity island in CRC genesis (Pleguezuelos-Manzano et al., 2020).

The efficacy of generating PSC-based cancer models from patients may depend on cancer type and on the efficiency on standardized differentiation protocols, including epigenetic reprogramming, used to obtain the cell type that corresponds to the cancer of interest. Hence, whenever feasible, it seems to be more practical to grow tumoroids directly from tSCs than to involve an intermediate PSC step. However, the ability of hPSCs to progress to defined differentiation states within developmental lineages, for which tSCs might be difficult to obtain, represents an unprecedented opportunity for cancer researchers, especially for pediatric cancers with their strong spatiotemporal developmental valence (Filbin and Monje, 2019). As an intermediate strategy, although inefficient compared with that of normal cells, generating iPSCs from a variety of cancer cell types has proven effective in the laboratory (Papapetrou, 2016). These cultures are then re-differentiated along the relevant lineage (from which the putative cell-of-origin arose) and retain an intact oncogenic cancer cell genome, in order to identify means of overcoming the differentiation blockade that often characterizes tumoral growth. In that context, concomitant reprogramming of isogenic normal cells for direct comparison is essential and can reveal how cancer genomes affect progression through cellular lineages (Stricker et al., 2013; Kotini et al., 2015). This approach is particularly advantageous when interrogating cancer-related processes addressing complex genomic aberrations that cannot be easily introduced through exogenous means, including copy number alterations and loss of genomic regions containing multiple genes and regulatory sequences. Nevertheless, it should be noted that the reprogramming of cancer cells into iPSCs themselves might be affected by the presence of somatic mutations and alter, *per se*, the lineage progression of cancer-iPSCs during directed differentiation, thus, confounding our understanding of such processes. This could particularly hold true for pediatric cancers due to their strong epigenetic component and to the importance of this particular chromatin context in the oncogenic reprogramming.

##### Top–down approaches: from tumoroids to oncogenic mechanisms

Experimentation relying on primary tumor samples has been hampered for a long time by difficulties to acquire enough material for large-scale studies. The situation is particularly complicated in the field of pediatric oncology. Indeed, unlike adult cancers, childhood cancers are rare (Waldron et al., 2010), and the collected samples are most often micro-biopsies aimed primarily at diagnosis to limit pain and preserve the integrity of the developing organism.

**FIGURE 4:**
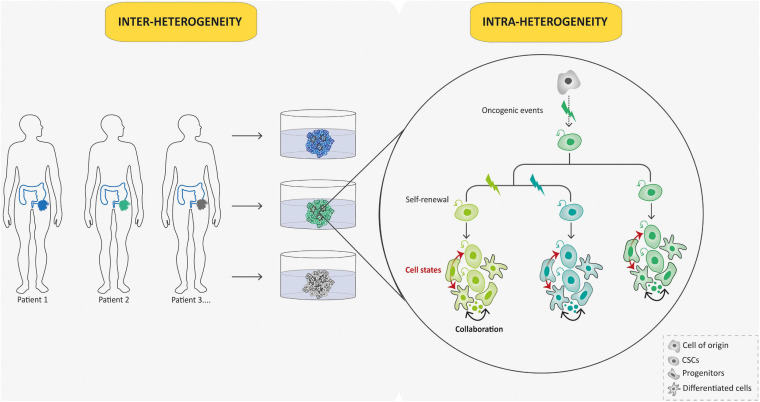
Tumoroid reconstruct inter- and intra-tumoral hierarchy and dynamics. Tumoroids can be used to evaluate the importance of tumor heterogeneity: inter-tumoral heterogeneity in which tumors of the same type but from different patients have distinct clinical features, and intra-tumoral heterogeneity in which different populations within a tumor have divergent genotypes and phenotypes. Indeed, in this case, different subclones (represented by different colors) emerge due to multiple oncogenic events from a common ancestor (cell-of-origin). Cancer stem cells (CSCs) that arise from these events can self-renew and produce various cell lineages present in a tumor (different cell states from each subclone are represented in respective colors). This intra-tumoral heterogeneity can be affected by the tumor microenvironment and collaboration between tumor cells.

Patient derived-tumoroids with their ability to be expanded *in vitro* while preserving many features of primary tumors offer an interesting opportunity to perform studies requiring both high sample quality and quantity. In contrast to the bottom–up approach, patient-derived tumoroids arising from human patients can be directly established from tumor needle biopsies (Nuciforo et al., 2018), surgical resections (Broutier et al., 2017), or ascitic and pleural fluid (Hill et al., 2018) to perform “top–down” studies of pre-established malignancies. Then, they appear of particular interest for rare cancers, such as pediatric ones, as they allow for the generation of large collections of living material for research purposes, despite the scarcity and small tumor sample sizes. From the first described colorectal cancer patient-derived tumoroid model developed by Sato et al. (2011; Figure 1), many others have emerged in the past decade, from multiple cancer types, including brain cancers (Jacob et al., 2020), almost all endoderm-derived tissue cancers [colon (van de Wetering et al., 2015), rectum (Ganesh et al., 2019), stomach (Vlachogiannis et al., 2018), esophagus (Li et al., 2018), pancreas (Boj et al., 2014), bladder (Mullenders et al., 2019), gallbladder (Saito et al., 2019), liver (Broutier et al., 2017), lung (Kim et al., 2019), and from gender-specific cancers [prostate (Beshiri et al., 2018), ovary (Nanki et al., 2020), endometrium (Boretto et al., 2019), and breast (Sachs et al., 2018)]. Of note, patient-derived tumoroids often grow at slower rates than their matching normal organoid counterparts probably due to higher rates of mitotic failure and subsequent cell death (Drost et al., 2015) and this can preclude their derivation. This drawback can be bypassed by using pure tumor cells as starting material whenever possible. The overgrowth of tumoroids by healthy organoids derived from normal tissue present in the tumor samples can also be avoided using selective culture conditions (Sato et al., 2011) or clonal culture condition and phenotypic selection (Broutier et al., 2017). Importantly, the studies cited above have all demonstrated that tumoroids preserve both histological and molecular (genomic, transcriptomic, and epigenomic) features of the parent tumor. This results in the robustness and the relevance of such models, since the significant heterogeneity among patient tumors, the so-called “inter-tumoral” heterogeneity, and within tumors themselves, the so-called “intra-tumoral” heterogeneity, are key limitations to studying cancer patient-specific attributes and perform relevant translational studies (Figure 4). Inter-tumoral heterogeneity refers to differences between patients having the same type of tumors (Figure 4). These tumor subtypes have specific individual molecular signatures, different biological behaviors, and, as a result, have a different impact on clinical outcome. Patient-derived tumoroids have the potential to provide connections between single patient-level genetic abnormalities and the biological tumor behavior. For example, using gastric cancer (GC) tumoroids, a recent study demonstrated that divergent genetic and epigenetic routes can lead to Wnt and R-spondin niche independency underlying the validity of tumoroid genotype-phenotype screening strategies in gaining further insight into human cancers (Nanki et al., 2018). Intra-tumoral heterogeneity refers to the coexistence of different malignant cell populations within a tumor (Figure 4). It is tightly connected to tumor evolution that depicts changes in intra-tumoral heterogeneity along the temporal axis. Indeed, cancers are presumed to originate from a single cell, and from there, tumoral evolution describes the dynamics by which subpopulations of cancer cells acquire genetic and phenotypic differences (Figure 4). Patient derived-tumoroids allow biomass expansion from single cell-derived clones, thus increasing the fidelity of whole-genome sequencing and enabling the extension to multi-omics sequencing to examine mutational processes in tumor tissue. Consistently, Roerink et al. (2018), using CRC-tumoroids obtained from multiple single cells, recently showed that CRC cells display an extensive intra-tumoral diversity of mutations, and DNA methylation and transcriptome states compared with normal colorectal cells. Another level of intra-tumoral heterogeneity depicts the hierarchically organized tumor cell community (from CSCs to more differentiated cells; Figure 4). The CSC concept states that, by analogy with the renewal of healthy tissues, a subset of tumoral cells, the CSCs, a self-renewing subpopulation of cancer cells, fuels tumor growth (Nowell, 1976; Clarke et al., 2006; Vermeulen et al., 2008). So far, therapy resistance and tumor relapse after drug therapy are commonly explained by Darwinian selection of pre-existing drug-resistant, often stem-like, cancer cells (Bao et al., 2006). Thus far, the existence of human CSCs is mainly supported by xenotransplantation dilution assays (Quintana et al., 2008) but their clonal dynamics and plasticity remain unclear. Moreover, the analysis of cellular hierarchies in human cancers has been hampered by the impossibility of labeling and tracking tumor cell populations in an intact environment. To overcome these limitations, owing to the robustness of organoid technology, several groups have devised strategies based on editing the genomes of patient−derived tumoroids using CRISPR/Cas9 technology to integrate reporter cassettes at desired marker genes. These genetic experiments confirm that human CRCs adopt a hierarchical organization reminiscent of that of the normal colonic epithelium, in which LGR5^+^ colorectal cancer cells serve as CSCs in growing cancer tissues (Cortina et al., 2017; Shimokawa et al., 2017). Remarkably, although elimination of CSCs by specifically targeting LGR5^+^ cells initially resulted in the reduction in primary tumors, this did not induce long-term regression of primary tumors, which instead displayed a dramatic plasticity with other Lgr5^–^ cells replenishing the LGR5^+^ CSC population (Shimokawa et al., 2017). These data provide insights into the plasticity of CSCs and may have broader applications to study cell heterogeneity in human tumors and implications in therapeutic cancer management. Because they allow to model the tumor cell hierarchy in its dynamic dimension, tumoroids therefore appear to be essential tools for understanding the contribution of functional heterogeneity and cellular plasticity in treatments resistance in pediatric field.

Aside from cancer modeling, organoid technology is a powerful tool to evaluate the efficacy and toxicity of drugs and precision treatment strategies. Indeed, with their rapid expansion and their ability to faithfully mimic both inter- and intra-tumoral heterogeneity, patient-derived tumoroids bridge the gap between traditional cell-line-based and primary sample-based methods. Thus, several groups have now used patient-specific tumoroids as platforms for functional testing such as drug screening and for correlating such data with the genetic make-up of individual tumors. Precision oncology, aiming at exploiting predictive biomarkers to orientate cancer therapeutic management, could benefit greatly from patient-derived tumoroid technology. Indeed, molecular profiling of such tumoroids may reveal causal tractable molecular changes underlying drug resistance, which could be used to stratify individual patients to specific treatment regimens. In the past years, patient-derived tumoroids have demonstrated their ability to identify new therapeutic strategies *via* the discovery of gene-drug interactions, thus correlating therapeutic vulnerability to archetypal genetic alterations. For instance, CRC tumoroids were used to investigate by phenotypic drug screening the effect of different RAS inhibitors, either as single agents or in combination. Interestingly, these treatments solely forced tumoroids into cell cycle arrest rather than cell death, and cells rapidly re-initiated growth when the treatment was stopped, thus, questioning the effectiveness of such therapeutic strategies in the treatment of *RAS*-mutant CRC (Verissimo et al., 2016). A recent study using breast tumoroids revealed PARP inhibition as a synthetic lethality of breast cancer with BRCA1 or BRCA2 mutational signatures (Sachs et al., 2018), while others revealed that tumor cells harboring gain-of-function mutations in FGFR3 exhibited significant sensitivity to the MEK and ERK inhibitors by using bladder tumoroid models (Lee et al., 2018). In the same way, prostate tumoroids have revealed that prostate cancer-associated SPOP mutations confer resistance to BET inhibitors through the stabilization of BRD4 (Dai et al., 2017), enabling a deeper understanding of how prostate cancers of certain patients respond to treatment according to their genetic lesions. The development of drug screening methods in patient-derived tumoroids is at the dawn of its development, but current nascent efforts for medium-scale drug screens on tumoroid biobanks have yielded promising results about the predictive value of tumoroids for individual drug responses and highlighted the pan-cancer type applicability of this approach. In pancreatic tumoroids, eight of the nine tumoroids responded similarly to the cognate patients when exposed to chemotherapy, giving a match rate of 89% (Tiriac et al., 2018). In the same study, authors demonstrates that tumoroid-based gene expression signatures of gemcitabine chemosensitivity enabled longitudinal assessment of chemosensitivity in patients and were correlated with improved progression-free survival in a 55-patient cohort (Tiriac et al., 2018). Along the same line, gastrointestinal tumoroids (CRC and gastroesophageal cancer) were generated and used to determine whether they could predict patient treatment response by assessing *in vitro* drug sensitivity and comparing with the actual response of patients (Vlachogiannis et al., 2018). The authors reported a positive 88% predictive value and a 100% negative predictive value (predicting that a particular drug does or does not work, respectively), suggesting that patient-derived tumoroids recapitulate patient responses in clinical trials and could be used for decision-making processes of early-phase clinical trials (Vlachogiannis et al., 2018). Moreover, a large living biobank of 80 tumoroids was recently derived from locally-advanced rectal cancer (LARC) enrolled in a phase III clinical trial. These tumoroids correctly predicted outcome in 84% of patients implying that they could be used in a clinic to predict LARC patient responses and may represent a companion diagnostic tool in rectal cancer management (Yao et al., 2020). Drug screening in patient-derived tumoroids may also help to identify unexpected treatments. For example, evaluation of gastric tumoroids from 34 patients, capturing regional heterogeneity and subclonal architecture, against a 37-compound library revealed sensitivity to unexpected drugs that were recently approved or in clinical trials, including napabucasin and abemaciclib (Yan et al., 2018).

**FIGURE 5:**
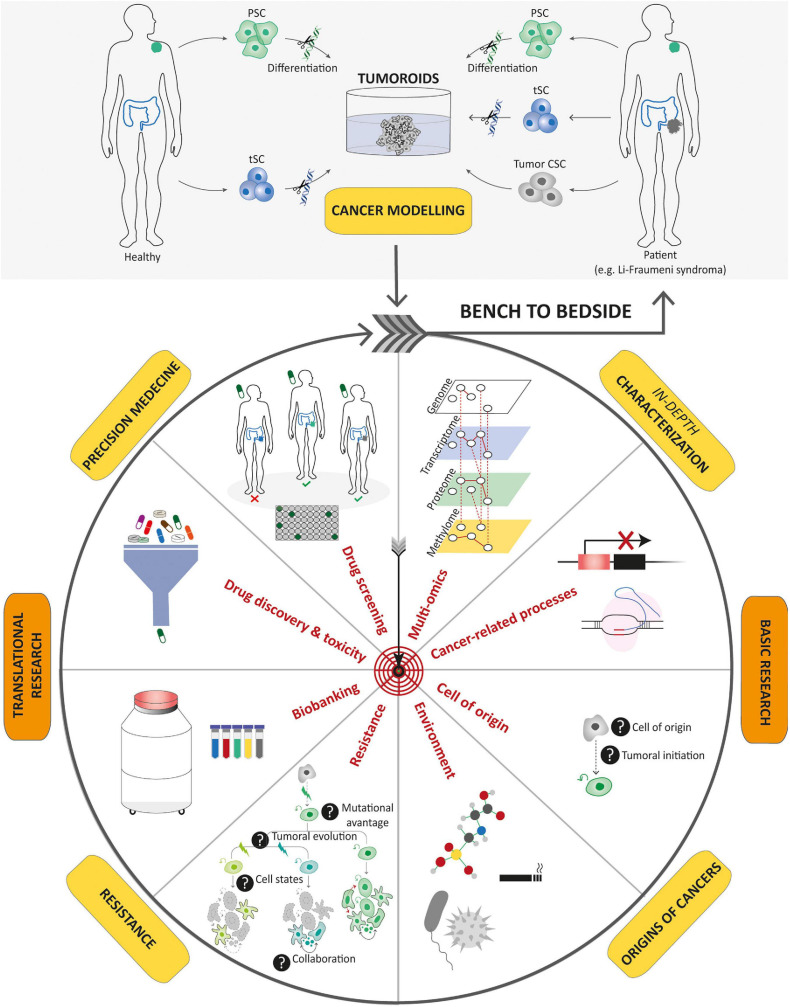
Potential applications of tumoroids in the field of cancer research. Tumoroids can be derived from PSCs and tissue-specific SCs after introduction of cancer-associated genetic alterations, or directly from tumor samples. These resulting tumoroids represent cancer models and can be profiled by multi-omics integrative analyses to decipher new oncogenetic processes. In basic research, tumoroids can be used to study cancer initiation and its related processes such as the understanding of the cell-of-origin and the links between tumorigenesis and infectious agents or environmental factors. In addition, tumoroids can be used to identify the biological underpinnings of tumor progression and resistance to treatments. Biobanks of tumoroids, in which samples obtained from patients are stored as a resource for future research, can promote the discovery of new cancer drugs and guide optimized therapeutic strategies for an individual or group of stratified patients by predicting drug responses. To conclude, tumoroids have the potential to translate scientific knowledge from bench to bedside with scientific discoveries being swiftly returned to the patient.

Patient-derived models are crucial for both basic and translational cancer research, and their roles will increase in the coming era of post-genome medicine (Figure 5). In the past century, conventional models have offered useful insights into cancer research and have guided the design of innovative candidate drugs, but they are not always developed for rare cancers. This creates a vicious circle for rare cancers such as pediatric ones, with the lack of effective therapies that might, in part, be attributable to the rarity of adequate patient-derived cancer models available for pre-clinical studies. Due to their intrinsic characteristics presented above, tumoroids definitely appear to be essential to meet the current challenges in pediatric oncology.

## Organoid Technology: A Powerful Tool to Unravel the Biology and Therapeutic Vulnerabilities of Pediatric Cancers

Recent success in tumoroid generation provides an appealing new bridge between basic and translational cancer research and is expected to gain momentum in the coming years as an essential tool to gain novel insights into tumor origin, initiation, progression, and treatment. Though largely applied to adult cancers, this technology is scarcely used for childhood cancers, with a notable delay in technological transfer. Indeed, to date, compared with dozens in adult oncology field, only three protocols have been described to grow pediatric patient-derived tumoroids (Saengwimol et al., 2018; Calandrini et al., 2020; Saltsman et al., 2020). Then one of the key challenges in the organoid field will be to extend this approach to non-epithelial tissues since pediatric cancers are mostly anything but carcinomas. In the following part, we will discuss why and how using tumoroids could revolutionize our understanding of pediatric cancers hence our ways to treat them.

### Unraveling the Developmental Origins of Childhood Cancers

**FIGURE 6:**
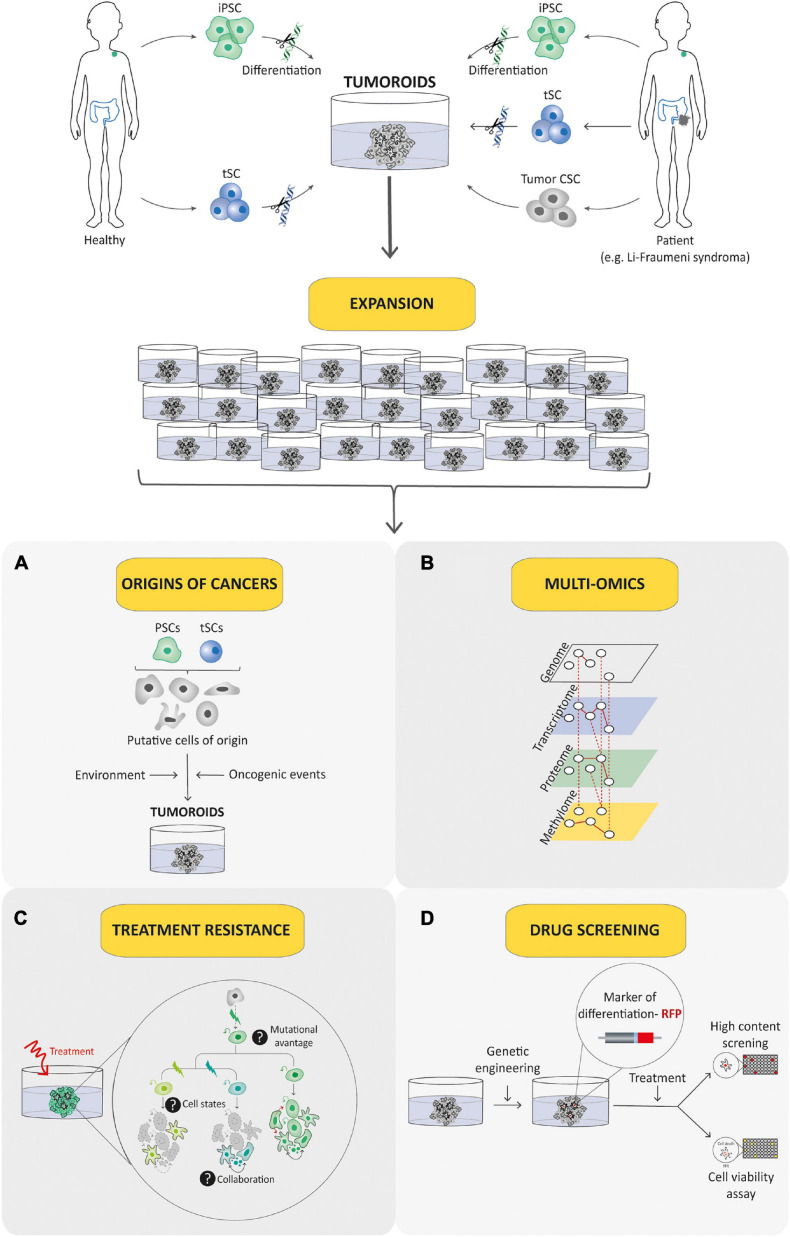
Tumoroid as a promising tool for understanding and treating pediatric cancer. Tumoroid technology can be exploited to model germline pediatric cancer predisposition syndrome, to study origins of cancers **(A)** and to perform multi-omic profiling analyses **(B)** in order to promote the understanding of pediatric cancer biology. In addition, tumoroids can be used to identify the biological underpinnings of cell death resistance such as tumoral cells state dynamics and collaboration **(C)** and to perform drug-screening analyses based notably on phenotypic screens **(D)** to explore innovative and powerful therapeutic possibilities.

Developmental origins of childhood cancers are supported by several features (Marshall et al., 2014). First, the unique age spectrum of most pediatric cancers suggests that there are time- and tissue-specific windows of susceptibility to cell transformation (Filbin and Monje, 2019). This observation has been summarized by Dyer and colleagues under the concept of “cellular pliancy,” which state that unique features of each cell type determine whether it will be susceptible, or not, to malignant transformation after sustaining a particular genetic alteration (Chen et al., 2015). Second, pediatric cancers mainly carry alterations in epigenetic factors (Gröbner et al., 2018) and developmental signaling pathways such as Notch, WNT, or Hedgehog and TGF-beta (Gröbner et al., 2018). Additionally, the theme of early development is also apparent in the strong correlation between developmental syndromes, such as RASopathies, neurofibromatosis type 1 (*NF1*) or Li-Fraumeni syndrome (LFS; *TP53*), and pediatric cancer predisposition (Zhang et al., 2015). Thus, any attempt to fully understand the origin of these cancers must consider not only the spectrum of molecular lesions but also the developmental stage of the tissue lineage in which they occur and the microenvironment in which the tumor eventually arises. As an example, rhabdoid tumors are highly aggressive pediatric tumors genetically quiet except for germline or somatic mutations of *SMARCB1*, a member of the SWI–SNF chromatin remodeling complex, functioning as a tumor suppressor in a broad range of developing tissues (Versteege et al., 1998). In mouse experiment, early embryonic *SMARCB1* inactivation between E6 and E10 induced rhabdoid tumors that closely resembled all transcriptional subclasses of human tumors. Interestingly, *SMARCB1* inactivation during slightly later embryonal stages (E12) did not lead to tumor formation (Han et al., 2016). These findings suggest that the *SMARCB1* mutation is tumorigenic only during a rather limited window during mouse brain development. Moreover, in these studies, mutational event and tumorigenesis appear to be temporally distinct, with mutation occurring in the early prenatal stage and delayed tumor manifestation in the early postnatal period pointing toward an important difference between the “cell-of-mutation” and the “cell-of-origin,” and underlining the need to better understand the microenvironmental factors influencing cell state during development. High-grade gliomas (HGG) of childhood and adolescence present a spatio-temporal pattern of incidence broadly mapping onto developmental waves of myelination in the human central nervous system (Gibson et al., 2018). Like for rhabdoid tumors, the cell-of-mutation may be disconnected from the cell-of-origin, since transformation probably occurs in a different developmental time frame. Indeed, recent evidence in mouse suggests that the classic genomic alterations associated with HGG only induce tumors in the postnatal period if introduced during prenatal brain development (Pathania et al., 2017). To fully elucidate the notion of cell-of-mutation and cell-of-origin in pediatric cancers, future studies in which the mutations are introduced at various developmental time points and in various cellular state and microenvironment contexts will be required. If cell identity and environment is a determinant of tumorigenic potential, it can also be a consequence of oncogenic activity and, as such, confound our understanding of origin of cancers. This was recently exemplified in the pediatric cancer field by a study showing that perturbations in the hedgehog pathway in mouse in the endothelial lineage led to rhabdomyosarcoma, pediatric cancer with skeletal muscle features (Drummond and Hatley, 2018). In that case, endothelial cells must provide a transcriptional program permissive for the tumorigenic effects to distort their cellular identity. Future efforts toward mechanistic understanding of developmental origins of pediatric cancers will therefore need to consider, on top of genetic alterations, the various pre- and postnatal developmental cell stages, their specific microenvironmental context, and their epigenetic landscape to solve the spatio-temporal and spatio-molecular patterns inherent to pediatric cancers and ultimately design dedicated innovative therapies. In that context, bottom–up approaches using organoid technology appear as a potential leveraging tool to succeed in such a challenging experimental set-up. Indeed, there are several challenges that have limited the creation of human cancer models purely through genetic engineering of otherwise normal human cells. Notably, many childhood cancers are thought to arise during pre-natal life, meaning that the relevant stem and progenitor cell populations are often unknown or difficult to obtain, which complicates efforts to build relevant organoid and subsequent tumoroids models. hPSC technologies can therefore play a decisive role to overcome this barrier, by facilitating access to a range of lineage and differentiation states to serve as platforms to build such models (Figure 6). Moreover, iPSC-derived organoids may be particularly useful in modeling germline cancer predisposition syndromes as exemplified by iPSC-derived brain tumoroid models developed to study the predisposition syndrome NF1 (Anastasaki et al., 2020). They could be directly derived from the patient with such syndrome as shown by a recent study on LFS revealing a role of impaired T53 signaling in defects of the imprinted gene network regulating osteoblast differentiation that may contribute to osteosarcoma development in LFS patients (Figure 6).

### Integrative Profiling of Pediatric Tumors to Decipher Pediatric Cancer Biology

Over the past 10 years, a tremendous effort has been made to define the genomic landscape of pediatric cancers. This led to groundbreaking discoveries including striking examples of genomic alterations that underscore marked differences between childhood and adult cancers. Notably, pediatric cancers exhibit fewer somatic mutations on average than adult cancers with an over-representation of transcription factor fusion oncogenes compared with adult tumors. These findings have fueled initiatives to develop pediatric cancer-specific therapies and to provide guidance for precision medicine treatment of individual patients. Precision medicine is based on genomic profiling of a given patient’s tumor, yielding information that can then be used to stratify patients based on molecular signatures and select therapies designed to counter the effects of specific driver mutations. The approach has had some successes, including the treatment of pediatric cancers with NTRK gene rearrangements (Laetsch et al., 2018). The identification of the Pax3/7-Foxo1 translocation as a high-risk marker in rhabdomyosarcomas makes it possible to envisage a de-escalation of treatments in fusion-negative entities, which is extremely important to limit the side effects of treatments in pediatrics (Nguyen and Barr, 2018; Chen et al., 2019; Pappo and Gartrell, 2020). In addition to genomics, a lot of progress has been made in the regulatory environment and the pharmaceutical industry, enabling cooperative trials of precision medicine, such as the Zero Childhood Cancer initiative in Australia, largest study to date, illustrating both the promise and challenges of the precision medicine approach (Wong et al., 2020). In this study, using a combination of tumor and germline whole-genome sequencing and RNA sequencing, authors analyze more than 250 tumor specimens from high-risk pediatric patients with cancer, and identified 968 reportable molecular aberrations. Specific therapeutic recommendations could be made and efficiency followed-up for about two-thirds of the patients (38 patients) among which only 31% exhibited a complete or partial response (Wong et al., 2020). This suggests that as for adult cancers, long-term benefits of this “sequence tumor and choose an agent” paradigm may ultimately apply only for a small percentage of patients (Marquart et al., 2018). Even though systemic sequencing of pediatric cancers will ultimately provide a more complete catalog of germline and somatic mutations in pediatric tumors and contribute to important insights into tumor heterogeneity and clonal evolution of pediatric cancers, it is foreseeable that these genomic and transcriptomic analyses alone will not be sufficient to define individualized therapies for each patient. Indeed, for the vast majority of pediatric patients with solid tumors, there are no somatic mutations in the tumor that can be exploited with the current arsenal of targeted therapies, and transcription factor fusion oncogenes have, thus far, been refractory to targeting (Nguyen and Barr, 2018; Chen et al., 2019; Pappo and Gartrell, 2020). Furthermore, the presence of a druggable mutation in a tumor and the availability of a drug that targets the dysregulated pathway does not ensure efficacy in patients, as exemplified by the inefficacy of anti-RAS therapies in *RAS* mutant rhabdomyosarcomas (Chen et al., 2019). The reasons for the current disillusionment are several, but accumulating evidence suggests that one of the biggest challenges is the difficulty to predict the biological impact of specific mutations. Thus, the lower mutational burden of pediatric tumors should not be confused with simplicity in the intrinsic molecular mechanisms of pediatric oncogenesis. Indeed, despite the extensive knowledge generated through recent genomic sequencing efforts, the functional and clinical implications of this knowledge have so far been limited, underlying the need of robust and relevant cancer models such as tumoroids to help in unraveling the biology and therapeutic vulnerabilities of pediatric cancers. Even beyond that, it is now well admitted that to study complex biological processes, genetics is not enough; it is imperative to take an integrative approach that combines multi-omics data to highlight molecular intricacy and variations at multiple levels such as genome, epigenome, transcriptome, proteome, and metabolome. To do so, accounting for the scarcity and the small size of samples in the pediatric oncology field, there is an ongoing need for close-to-native patient-derived cancer models that could be fulfilled by patient-derived tumoroid models. Indeed, they could overcome the limitation of tumor material available for omics analyses, since they are sufficiently amplifiable and thereby amenable to molecular, proteomic, and metabolic profiling (Lindeboom et al., 2018; Figure 6). Following this strategy, the Multi-omics and Organoid Screening Program, part of the MPCCC (Monash partners comprehensive cancer consortium) Precision Oncology program, is a pilot study designed to extend molecular profiling of patient tumors beyond genomic characterization to integrated multi-omics and functional analyses^1^. By generating integrated, multi-omics, and personalized cancer profiles, this initiative aims at better informed treatment options for cancer patients, and where possible, to recommend personalized treatment plans. To ensure that this laboratory-based research will translate to the benefit of cancer patients, results of the multi-omics and organoid screening will be discussed by scientists and research-active cancer clinicians. By serving as characterization tools for pediatric cancers on a multi-omic scale, tumoroids could make it possible to apprehend the specific deregulations of each tumor at different biological scales and no longer base precision medicine choices solely on genomic/transcriptomic characterization.

### Understand Pediatric Cancer Heterogeneity and Plasticity to Conquer Resistance to Treatments

During development, cell specification is engaged by cell type-specific transcription factors. The establishment and maintenance of these transcriptional programs rely on cell type-specific patterns of chromatin organization and dynamics, making epigenetics a key element in the regulation of cell fate. Accumulating evidence suggests that pediatric tumors could result from direct dysregulation of cellular specification during development or co-option of epigenetic developmental programs through pre- and postnatal cell reprogramming. Moreover, it has been widely presumed that differentiated cells are determined during development and become irreversibly committed to their designated fates. However, in certain circumstances, differentiated cells can display plasticity by changing their identity, either by dedifferentiation to a progenitor-like state or by transdifferentiation to an alternative differentiated cell type. By analogy with physiological contexts, in which cellular plasticity allows cells to respond to external stresses and adapt to their environment, cellular plasticity may represent a cardinal feature of tumoral cells to evade treatments or escape the confines of the primary tumor. Thus, alterations in epigenetic processes involved in cell specification and plasticity appear to be central in pediatric oncology. With the recent democratization of single-cell RNA-sequencing, increasing evidences support that pediatric cancers are hierarchically organized tumors recapitulating impaired developmental trajectories and possessing CSC responsible for maintenance, relapse, and metastasis of the tumor (Couturier et al., 2020; Gojo et al., 2020; Kildisiute et al., 2021). Then, our ability to reconstruct intratumoral hierarchy and dynamics in pediatric cancer models is therefore crucial for understanding pediatric cancer biology. However, up to now, intra-tumoral heterogeneity and the mechanisms behind it remain a black box in the field of pediatric oncology, mostly again as a result of scarcity of tumor material and considering that chemotherapies are most often administrated before surgery to children and adolescents. Tumoroids then represent again a promising approach for creating patient-derived *in vitro* cancer models that closely recapitulate the overall pathophysiological features of natural pediatric tumorigenesis and allow to have a sufficient quantity of material available. Indeed, as discussed previously, they are highly relevant cellular models to study non-genetic cell state dynamics associated with resistance to treatment for exploring new therapeutic possibilities, since they mimic cellular intra-tumoral heterogeneity by preserving the differentiation hierarchy (from CSC to more differentiated cells; Figure 6). In that context, organoid technology conjugated to genetic engineering, light microscopy (i.e., confocal imaging or light-sheet technology available in the laboratory), and orthotopic transplantation appears as a powerful approach to probe the human cancer cellular dynamics *in vitro* and *in vivo*, as recently exemplified in colorectal cancer (Cortina et al., 2017; Shimokawa et al., 2017). In addition, they are a more amenable system for the manipulation of niche components, signaling pathways, and genome editing (e.g., CRISPR/Cas9) than *in vivo* models. Then, they provide an unprecedented way to unravel the dynamics of tumoral cells and notably individual CSC. Recently developed lineage-tracing and cell-ablation strategies in tumoroid systems have provided insights into CSC plasticity, and evidenced that both CSC and non-CSC are plastic and capable of undergoing phenotypic transitions in response to appropriate stimuli (Batlle and Clevers, 2017; Cortina et al., 2017; Shimokawa et al., 2017). The preponderance of epigenetic alterations in pediatric cancers probably reflect a locked-in epigenetic state during development, which contributes to the final gene expression and phenotype of the resulting cancers. Then, the possibilities of genetically engineered tumoroids to express fluorescent reporter of cellular differentiation state could open up a new avenue for phenotypic drug screening of agent susceptible to bypass the default lineage differentiation processes observed in most pediatric tumors in order to terminate malignant proliferation by engaging terminal differentiation (Figure 6). Such approaches should go beyond classical drug screening and help pinpoint cellular mechanisms of resistance to treatment link to tumoral cell states.

### Clinical Trials on Children, a Challenge to Overcome

Although outcomes for children with cancer have significantly improved over the past 50 years, there has been little progress in the treatment of some pediatric cancers, particularly when in an advanced stage. Moreover, children are not little adults. However, treatments for pediatric cancers are most often variations of those for adults, which led to delusions as with immunotherapies. In fact, there are many examples of differences in the response to therapies when comparing childhood cancers to their histological counterpart in adult cancers. For example, temozolomide, an alkylating agent that has demonstrated survival benefit for adult HGG (Stupp et al., 2005) exhibits no clinical benefit for pediatric gliomas (Cohen et al., 2011). Thus, childhood cancer targets may be non-overlapping with those in adult malignancies notably because of actively developing tissues context in which these cancers arise, suggesting the need of dedicated therapeutic agents and administration program. Additionally, clinical trials are often insufficient to conduct clinical pharmacology studies in children, due to many challenges such as the very limited pediatric population. In that context, increasing efforts should be made to carefully model and predict with robust preclinical data the most promising drugs for children before new compound submission for clinical trials. Since they accurately recapitulate pediatric cancers and are easy to amplify, models such as pediatric patient-derived tumoroids will ultimately be necessary for the design of efficient, less toxic therapies adapted to children. To date, compared with dozens in adult oncology field, only three protocols have been described to grow pediatric patient-derived tumoroids underlying the urgent need to transfer this technology to children (Saengwimol et al., 2018; Calandrini et al., 2020; Saltsman et al., 2020). Recently, Drost and colleagues established and characterized malignant rhabdoid tumoroids that retain key properties of native tumors (Calandrini et al., 2020). Altogether, authors showed that rhabdoid tumoroids can be derived with high efficiency, rapidly expanded, and are amenable to gene editing, and allow for high-throughput drug testing to reveal patient-specific drug sensitivities (Calandrini et al., 2020). Parallel efforts to understand the complex mechanisms through which childhood cancer therapies disrupt normal tissue development, homeostasis, and plasticity are also needed to elucidate strategies to prevent long-term sequelae of childhood cancers (Pierson et al., 2016). In that context, organoids derived from non-tumoral tissues appear as an appealing strategy, to develop treatments more efficient, but also less toxic.

Thus, the opportunities exist to improve pediatric drug discovery and development efforts, and should be part of a collaborative interaction between academicians, pathologists, clinicians, drug developers, and health authorities.

## Discussion

Here, we reviewed how the unique attributes of the tumoroid models have been used in cancer research field to identify new mechanisms of tumor initiation, progression, relapse, and therapeutic strategies, and how they could further our understanding of pediatric oncology.

Pediatric cancer is a leading cause of death in children and adolescents, and this remains true despite the multiple international clinical trials conducted in recent years. Even worse, survival rates have not improved in the last 20 years or so. It, therefore, seems clear that the approach that consisted of transposing the therapies discovered for cancers from adults to children and adolescents is no longer sufficient. Despite the validation in trials of biological markers, diagnostic tools, and targeted drugs based on “omics” data in the last few years, we *do* need to unravel molecular underpinnings specifically driving childhood cancers to discover new therapies, to overcome resistance and prevent sequelae. This will necessarily involve the development of specific, dedicated models summarizing the complexity of pediatric tumors, and we have shown here that tumoroids are ideal candidates in many aspects.

One of the main challenges to design such pediatric tumoroids will be to transpose this technology to non-epithelial cancers, such as sarcomas or other cancers of mesenchymal origin, for example, that are more frequent in children and adolescents. However, the classical strategy based on reconstituting tumor microenvironment *in vitro* by working on culture conditions *via* deciphering active signaling cascades that could support tumor cell growth gives promising results for us and others. One of the pitfalls remains to be access to patients’ tumor samples, which are often micro-biopsies. The importance of coordinating clinical and research teams so as not to impact the diagnosis while allowing a sample fragment to be cultured is a major challenge to enable the oncology field to benefit from these innovative models. Similarly, sharing tumor sample sequencing and clinical data is crucial to allow the inclusion of patients in ongoing precision medicine clinical trials, while allowing research teams to verify the quality and suitability of their tumor models with the original tumors. Fortunately, current efforts of ongoing pediatric data collection and Pan-Cancer projects indicate future opportunities for childhood cancer research that are greatly needed for both basic as well as clinical research. These include public portals such as St. Jude Research Hospital’s one in the United States., which seeks to discover the genetic origins of childhood cancer and find new cures, and provide raw sequence data for all published results freely available to the global research community; or the initiative of the French researchers in pediatric oncology network React-4Kids, created in 2018 and which was recently awarded to perform a national multi-omics analysis on pediatric tumors at diagnosis vs. relapse shared freely upon request *(Share-4Kids, France)*. The current initiative developed by the Human Cancer Models Initiative^2^, an international consortium aiming at generating and providing as a community resource, the next-generation tumor-derived culture models informed with -omics and clinical data, should also participate to make the difference in the coming years.

The stakes are high because tumoroids appear to be ideal models to study how tumoral heterogeneity impact response to treatments, which has barely been investigated, so far, in childhood cancers. First, because they will depict inter-tumor heterogeneity, pediatric tumoroid models should enable us to understand why patients that apparently present the same clinical features respond differently to treatments, a key issue in therapeutic management. Moreover, they should be decisive to define how intra-tumor cell heterogeneity and plasticity likely play a role in resistance to treatments and relapse. Indeed, by allowing the amplification of tumor material while preserving its characteristics as closely as possible, tumoroids will allow unprecedented single-cell -omics approaches to unravel, at genetic-epigenetic-transcriptomic levels, the diversity of tumor cells before and after treatment. Moreover, the relative ease of genetically modifying them to express fluorescent reporters of specific tumoral sub-populations (Artegiani et al., 2020) should help to dissect and monitor over time by 3D imaging (Gonzalez et al., 2021) mechanisms identified as playing a causal role in tumor escape.

At the same time, the use of organoid models derived from healthy tissues will make it possible to define the identity of childhood cancers’ “cell-of-origin,” which is definitely a key issue to resolve spatio-temporal and spatio-molecular patterns involved in pediatric cancers. Moreover, organoids and tumoroids should allow us to study accurately the impact of environmental factors on tumor initiation and progression, respectively, thereby building a bridge between basic and epidemiological research on childhood cancers, the latter often complicated by the small size of the cohorts studied. Another major advantage of exploiting organoid technology in the field of childhood cancers is definitely the possibility to perform drug screening approaches. By combining the use of tumoroid and organoid models, which can often be grown from corresponding healthy tissue, they could offer an opportunity for screening the drugs that specifically target tumor cells while leaving healthy cells unharmed.

So will we be definitively out of the woods and able to understand the complexity of pediatric cancers with the development of such tumoroid and organoid models? Probably not, but we will undeniably have made progress.

The lack of stroma, blood vessels, and immune cells are an intrinsic limitation of organoid technology and may preclude universal applicability. Future studies should address the possibility of developing organotypic systems incorporating additional cellular elements. Recently air–liquid interface (ALI) culture system was developed to grow tumoroids that preserved the complex cellular diversity of both endogenous tumor and stroma cells such as cancer-associated fibroblasts, and infiltrating immune cells over 1 to 2 months (Neal et al., 2018). Another caveat is that tumor heterogeneity is a significant potential confounding variable for extrapolating therapeutic response from a single biopsy region. Indeed, tumors are often heterogeneous mass of cells, and this intratumoral heterogeneity is increasingly being recognized as a highly complex process with high clinical impact that deserves special attention from practicing pathologists and researchers when it comes to derive tumoroids from patient material (López and Cortés, 2017). Whenever possible, multi-site sampling and associated tumoroid derivation should be performed to represent faithfully the intratumoral heterogeneity existing *in vivo* in the full-size tumor.

**FIGURE 7:**
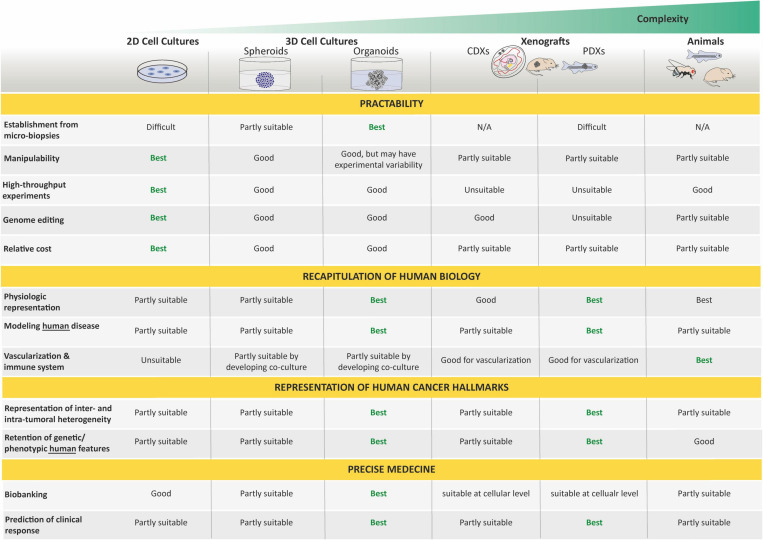
Strengths and weaknesses of organoids in cancer modeling. Organoids are assessed here for their relative benefits and limitations for cancer research by comparison with other model systems. Respective features are rated as best, good, partly suitable, and unsuitable. By bridging the gap between conventional 2D culture and animal models, organoids provide a unique opportunity to deal with a moderate system complexity meanwhile capturing the complexity of tumors. N/A, non-applicable.

The next challenge will be to define how to optimize these models so that they can also allow us to understand the impact of the ontogenic context on tumor initiation and escape, or resistance to treatment. Even though they are highly promising tools, tumoroids are unlikely to ever replace mouse experiments for such questions and even for drug screening. Compound hits will still optimally require verification in xenograft experiments, but this should be facilitated by the experimental tractability of tumoroids undergoing ready expansion and *in vivo* xenografting allowing a greater throughput than mouse PDX studies (Pauli et al., 2017). Nevertheless, studies comparing drug sensitivity patterns between sibling pairs of patient-derived tumoroids and mouse PDX models have been performed in CRC models (Pauli et al., 2017; Schütte et al., 2017) and showed different patterns for individual drug sensitivity in both models, probably because even though both models recapitulate many key features of their parental tumors, they also possess distinct selective pressure patterns and drawbacks (Bleijs et al., 2019). In a foreseeable future, studies combining the strengths of multiple preclinical models will probably appear as the best way to investigate therapeutic responses and provide clinically relevant metrics for precision and personalized medicine approaches (Figure 7).

How tumoroids will change the cancer treatment paradigm is then still uncertain. We may wish for them to herald an attractive new paradigm, but as researchers, we have to remember that wishing can confound our thinking. Acceptance of the cancer complexity should lead to humility as well as a healthy skepticism when confronting excessive promises. The aphorism attributed to the statistician George Box *“All models are wrong, but some are useful”* should be applied here to underline that, while tumoroids will certainly help us to address many of the current needs in cancer research, they are probably just a stepping stone toward something that we still need to imagine.

## Author Contributions

LB and VB wrote the manuscript. LB supervised the work. Both authors approved the manuscript for publication.

## Conflict of Interest

The authors declare that the research was conducted in the absence of any commercial or financial relationships that could be construed as a potential conflict of interest.

## Footnotes

1 https://www.monashpartnersccc.org/multi-omics-and-organoid-screening-program/

2 https://ocg.cancer.gov/programs/HCMI
